# *Vaccinium virgatum* Aiton Leaves Extract Suppressed Lipid Accumulation and Uric Acid Production in 3T3-L1 Adipocytes

**DOI:** 10.3390/plants10122638

**Published:** 2021-11-30

**Authors:** Masao Yamasaki, Yusei Kiue, Kento Fujii, Moe Sushida, Yumi Yamasaki, Kazuhiro Sugamoto, Yosuke Suzuki, Yasuko Koga, Hisato Kunitake, Hisahiro Kai, Kenjiro Ogawa, Kazuo Nishiyama, Yo Goto, Takayuki Nakayama

**Affiliations:** 1 Department of Biochemistry and Applied Biosciences, Faculty of Agriculture, University of Miyazaki, Miyazaki 889-2192, Japan; ykyk0246@gmail.com (Y.K.); kntfji1213@gmail.com (K.F.); gc18025@student.miyazaki-u.ac.jp (M.S.); gc18022@student.miyazaki-u.ac.jp (Y.K.); hkuni@cc.miyazaki-u.ac.jp (H.K.); nishiyam@cc.miyazaki-u.ac.jp (K.N.); 2 Faculty of Regional Innovation, University of Miyazaki, Miyazaki 889-2192, Japan; yamasakiy@cc.miyazaki-u.ac.jp; 3 Department of Applied Chemistry, Faculty of Engineering, University of Miyazaki, Miyazaki 889-2192, Japan; sugamoto@cc.miyazaki-u.ac.jp (K.S.); hg18031@student.miyazaki-u.ac.jp (Y.S.); 4 Department of Pharmaceutical Health Sciences, School of Pharmaceutical Sciences, Kyushu University of Health and Welfare, Nobeoka 882-8508, Japan; hkai@phoenix.ac.jp; 5 Organization for Promotion of Tenure Track, University of Miyazaki, Miyazaki 889-2192, Japan; ogawa.kenjirou.u2@cc.miyazaki-u.ac.jp; 6 Biolabo Co., Ltd., Hyogo, Kobe 650-0047, Japan; ygoto@ge-hd.co.jp (Y.G.); tnakayama@ge-hd.co.jp (T.N.)

**Keywords:** adipocyte, differentiation, uric acid, xanthine oxidoreductase, blueberry leaf

## Abstract

Blueberry (*Vaccinium virgatum* Aiton; Kinisato 35 Gou) leaves have recently attracted increasing attention as a useful material for the prevention of lifestyle diseases. Here, we examined the effects of the hot water extract of blueberry leaves (BLEx) on lipogenesis and uric acid production in 3T3-L1 adipocytes. The results showed that BLEx suppressed lipid accumulation and the mRNA expression of differentiation markers in 3T3-L1 adipocytes. A fractionation study showed that the highly polymerized proanthocyanidin-rich fraction was responsible for this effect. Upon maturation to adipocytes, 3T3-L1 cells produced uric acid and tumor necrosis factor-α, and hypoxia stimulated the production of uric acid and xanthine oxidoreductase activity. BLEx suppressed the production of uric acid under these conditions. Although BLEx inhibited the enzymatic activity of xanthine oxidase, this activity was observed in several fractions containing catechin, epicatechin, chlorogenic acid, rutin, and low molecular weight proanthocyanidins. Taken together, these results indicate that BLEx contains various compounds with the ability to suppress lipid accumulation and uric acid production in adipocytes.

## 1. Introduction

Obesity is an underlying condition associated with various lifestyle diseases and is an emerging problem worldwide. Utilizing functional foods for preventing obesity is a reasonable approach in daily life, and recent accumulating studies indicate its feasibility. Various plants and their extracts have been attracting increasing attention for the prevention of obesity and lifestyle diseases such as diabetes and nonalcoholic steatohepatitis [1,2,3]. Among these, we have focused on the blueberry *(Vaccinium virgatum* Aiton) leaf as a putative material for the prevention of lifestyle diseases. Blueberry leaf infusion has been used as a folk medicine for lifestyle-related diseases in Europe, even though scientific evidence proving its benefits has not been fully established. Actually, blueberry (*Vaccinium corybosum* L.) leaf extract had the ability to inhibit inflammatory response in mouse macrophage-like cells [4].

Recently, we have confirmed the safety of blueberry leaf hot water extracts (BLEx) in humans, and several blueberry leaf beverages and relevant items are commercially available in Japan. Further, Tetsumura et al. established a new breed of *Vaccinium virgatum* Aiton “Kunisato 35 Gou” for the cultivation of leaves [5]. The cultivation of Kunisato 35 is spreading in Miyazaki prefecture, in the southern part of Japan, and we have been evaluating the beneficial function of this breed. Results from animal studies showed that BLEx suppresses insulin resistance and reduces body fat in high-fat, high-sucrose-induced obese mice [6,7]. These papers also showed the inhibitory effect of BLEx on the hepatic lipid accumulation, and Li et al. showed that polyphenol in BLEx directly prevented lipid accumulation in HepG2 human hepatocytes [8]. Further, a recent human clinical study showed that beverages including BLEx suppressed the postprandial increase of triacylglycerols [9]. BLEx has a slightly sour and astringent taste because of the presence of organic acids such as quinic acid, and is rich in various polyphenols such as rutin, chlorogenic acid, catechin, epicatechin, and proanthocyanidins [10,11]. These polyphenolic compounds were considered to contribute to the strong antioxidant and anti-inflammatory properties of BLEx [12]. Although various beneficial effects have been observed with BLEx, specific active compounds have not been identified, especially in the prevention of lifestyle diseases.

Hyperuricemia is a major cause of gout and is drastically increasing in Japan. As lack of exercise and changes in dietary style are considered the reasons for the increase in gout, gout is closely associated with visceral fat obesity [13]. Xanthine oxidoreductase (XOR) is a key enzyme in uric acid synthesis that catalyzes the conversion of hypoxanthine to xanthine and then to uric acid. XOR comprises two different forms, xanthine dehydrogenase (XDH) and xanthine oxidase (XOD); the latter is involved in the excessive generation of reactive oxygen species such as superoxide and hydrogen peroxide, which results in the induction of tissue oxidative stress in a hyperuricemic state [14,15]. XOR is a medical target of gout remedies to reduce serum uric acid; allopurinol and febuxostat are the representative inhibitors of XOR, prescribed to patients with hyperuricemia. Elevation of XOR activity in the liver and adipose tissue has been observed in mice fed a high-fat diet, whereas XOD inhibition attenuated insulin resistance and diet-induced steatohepatitis in mice [16]. Notably, Tsushima et al. showed that Xor mRNA expression and XOR activity were elevated in visceral adipose tissue, but not in the liver and small intestine, of ob/ob mice compared with that in wild-type C57/Bl mice [17]. These reports indicate that XOR in adipose tissue is a critical target for the inhibition of uric acid synthesis in obese conditions. Accumulating research on the exploration of XOR inhibitors has revealed the importance of polyphenolic compounds [18]. The inhibitory effect of polyphenols on XOR activity depends on their chemical structure [19,20] and the degree of polymerization in the case of proanthocyanidin [21].

3T3-L1 cells are generally used as adipocyte models for evaluating cellular lipid accumulation. Further, accompanied with their maturation to adipocytes, 3T3-L1 cells can produce high levels of uric acid with elevation of XOR activity. In this study, we aimed to evaluate the effect of BLEx and its responsible components on adipogenesis and uric acid production in 3T3-L1 cells. The XOR inhibitory effect of BLEx was also evaluated to identify the components responsible for the reduction in uric acid synthesis.

## 2. Results

### 2.1. Effect of BLEx on Adipocyte Maturation

**Figure 1:**
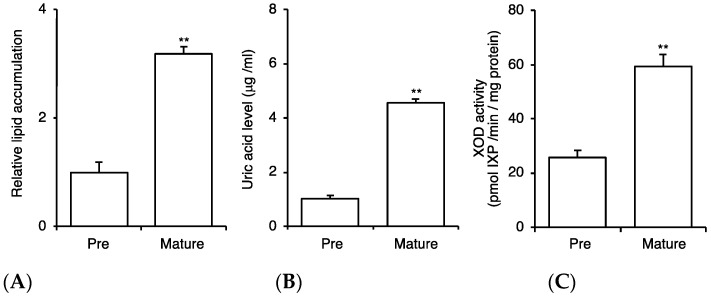
Comparison of adipogenesis and uric acid production from 3T3-L1 cells between the stages of preadipocytes and matured adipocytes. Data are means ± SE for 3 independent experiments. Asterisks indicate significant differences compared to the preadipocytes (pre) at *p* < 0.01, as evaluated by Student’s *t*-test. (**A**): Lipid accumulation evaluated by Oil Red O staining. (**B**,**C**): Uric acid release into media and cellular XOD activity.

**Figure 2:**
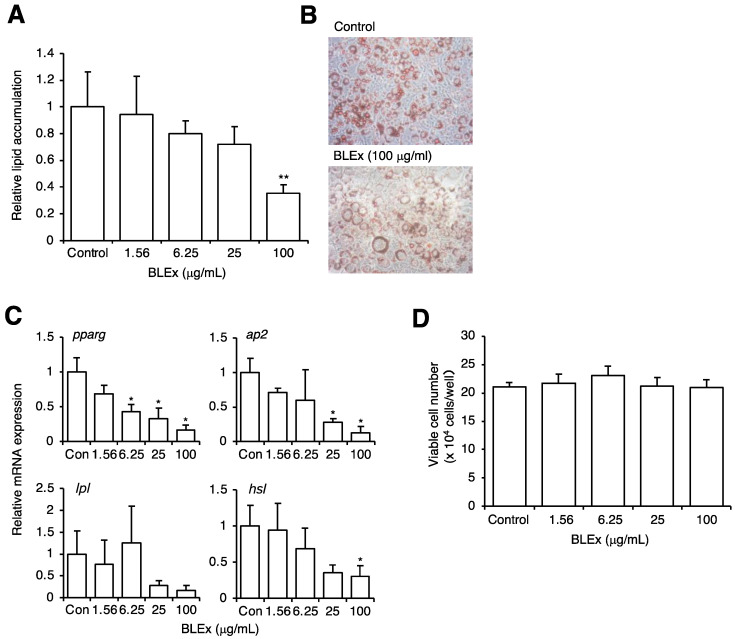
Effect of BLEx on the differentiation and maturation of 3T3-L1 cells to adipocytes. Data are means ± SE for 3 independent experiments. Statistical analysis was performed using one-way analysis of variance (ANOVA) and post hoc tests (Tukey–Kramer multiple comparisons). Asterisks indicate significant differences compared to the control at *p* < 0.05 * or *p* < 0.01 **. (**A**,**B**): Lipid accumulation was evaluated by Oil Red O staining and representative images are shown in B. (**C**): mRNA expression of adipocyte differentiation markers was measured by real-time PCR, and the data are shown as values relative to the mRNA expression of the internal control, *gapdh.* (**D**): Cytotoxicity of BLEx was evaluated as trypan blue exclusion method.

First, we confirmed the response of 3T3-L1 cells before and after maturation to adipocytes, as reported previously. We evaluated lipid accumulation, uric acid production, and XOD activity. All these parameters were prominently upregulated after maturation (Figure 1), verifying that this cell line was suitable for the simultaneous evaluation of adipogenesis and uric acid production. During the maturation of 3T3-L1 cells to adipocytes, the cells were treated with various concentrations of BLEx, and the lipid accumulation and expression of maturation mRNA markers were evaluated (Figure 2). The results showed that BLEx dose-dependently suppressed the accumulation of intracellular lipids, and that a significant difference was observed at 100 μg/mL (Figure 2A,B). We confirmed that BLEx did not show cytotoxic effect on 3T3-L1 cells at these given concentrations (Figure 2D). We evaluated the mRNA expression of *pparg*, *ap2*, *lpl*, and *hsl* as the maturation markers of adipocytes and found that BLEx suppressed their expression. Significant differences were detected in the expression of *pparg*, *ap2*, and *hsl* at 100 μg/mL.

### 2.2. Effect of Fractionated BLEx on Adipocyte Maturation

**Figure 3:**
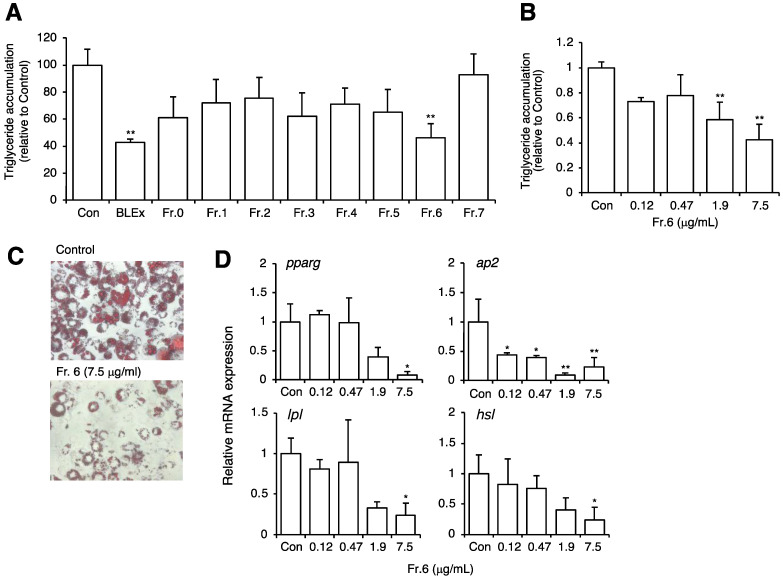
Effect of fractionated BLEx on the differentiation and maturation of 3T3-L1 cells to adipocytes. Data are means ± SE for 3 independent experiments. Statistical analysis was performed using one-way analysis of variance (ANOVA) and post hoc tests (Tukey–Kramer multiple comparisons). Asterisks indicate a significant difference compared to the control at *p* < 0.05 * or *p* < 0.01 **. (**A**–**C**): Lipid accumulation was evaluated by Oil Red O staining and representative images are shown in C. (**B**) shows the dose dependent effect of Fr. 6. (**D**): mRNA expression of adipocyte differentiation markers was measured by real-time PCR, and the data are shown as values relative to the mRNA expression of the internal control, *gapdh*.

To identify the active compound, BLEx was fractionated using Sephadex LH-20 resin by targeting the separation of polyphenols and proanthocyanidins. The effect of each fraction (Fr. 0–Fr. 7) on adipocyte maturation was then evaluated. The concentrations of each fraction, shown in Figure 3A, were determined from the respective concentrations contained in 100 μg/mL BLEx. Among the fractions, only Fr. 6 (7.5 μg/mL) showed the ability to suppress lipid accumulation (Figure 3A–C), and a suppressive effect on adipocyte maturation was also observed in terms of lipid accumulation and mRNA expression of maturation markers (Figure 3D).

### 2.3. Effect on Lipolytic Activity of Adipocytes

**Figure 4:**
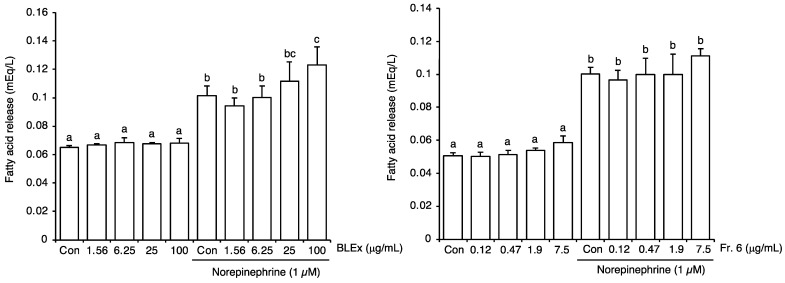
Effect of BLEx and Fr. 6 on the lipolysis of 3T3-L1 cells in matured adipocytes. Data are means ± SE for 3 independent experiments. Statistical analysis was performed using one-way analysis of variance (ANOVA) and post hoc tests (Tukey–Kramer multiple comparisons). Different alphabets indicate significant differences at *p* < 0.05. Matured adipocytes were stimulated with norepinephrine to promote lipolysis. Fatty acid release into the culture supernatant was measured as an index of lipolysis.

To evaluate the effects of BLEx and Fr. 6 on lipolysis, matured 3T3-L1 adipocytes were treated with norepinephrine, and fatty acid release was measured (Figure 4). Norepinephrine significantly stimulated the release of fatty acids into the medium. In this experiment, cells were stimulated with BLEx and Fr. 6 for 24 h before stimulation, whereas successive treatments with BLEx and Fr. 6 are shown in Figure 2 and Figure 3. Therefore, the effects of BLEx and Fr. 6 on differentiation and maturation could be negated in this experiment. Norepinephrine significantly promoted the release of fatty acids, but neither BLEx nor Fr. 6 affected fatty acid release without stimulation. Under norepinephrine stimulation, BLEx dose-dependently promoted fatty acid release between 25–100 μg/mL, whereas Fr. 6 had no effect.

### 2.4. Analysis of Proanthocyanidins

Our previous study indicated that Fr. 4–7 were rich in proanthocyanidins, and their structural composition was analyzed by thiolysis (Table 1). The proanthocyanidin content in Fr. 4, 5, 6, and 7 exceeded its level in BLEx; in particular, Fr. 5 and 6 contained 3.04- and 4.12-fold higher proanthocyanidins compared to BLEx, respectively. The proanthocyanidin levels in Fr. 0, 1, 2, and 3 were lower than those in BLEx (data not shown). We then focused on polymerization analysis using Fr. 4, 5, 6, and 7. The mean degree of polymerization (mDP) of proanthocyanidins increased with the fraction number, and the mDP of Fr. 6 was 7.55. The percentage of A-type bonds was similar among Fr. 4, 6, and 7 (4.4–5.8%), whereas that in Fr. 5 was significantly high (14.7%). The percentage of cinchonain-I units was similar among Fr. 5, 6, and 7 (3.8–6.6%), whereas that in Fr. 4 was significantly high (61.5%), which strongly influenced the low percentage of B-type bonds (33.3%).

### 2.5. Effect on Uric Acid Production from Adipocytes

**Figure 5:**
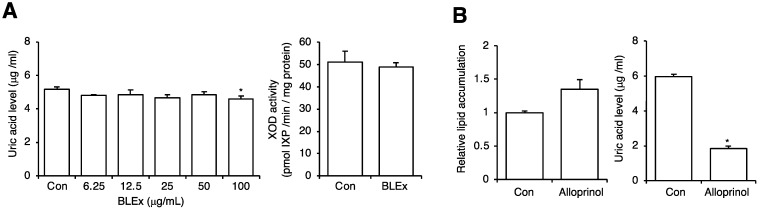
Effect of BLEx on the uric acid production of 3T3-L1 adipocytes. Data are means ± SE for 3 independent experiments. Statistical analysis was performed using one-way analysis of variance (ANOVA) and post hoc tests (Tukey–Kramer multiple comparisons) in the effect of BLEx on uric acid (most left panel) or Student’s *t*-test (other 3 panels). Asterisks indicate significant differences compared to the control at *p* < 0.05. (**A**): Effect of BLEx on uric acid release into the media and cellular XOD activity. (**B**): Effect of allopurinol, an inhibitor of XOD, on the lipid accumulation (Oil Red O staining) and uric acid release into the media.

**Figure 6:**
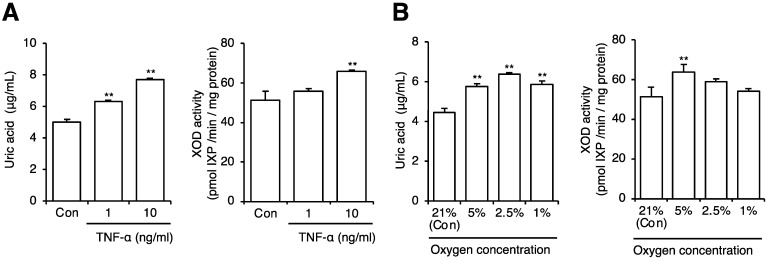
Effects of TNF-α and hypoxic stress and on uric acid production by 3T3-L1 adipocytes. Data are means ± SE for 3 independent experiments. Statistical analysis was performed using one-way analysis of variance (ANOVA) and post hoc tests (Tukey–Kramer multiple comparisons). Asterisks indicate significant differences compared to the control at *p* < 0.01. Matured adipocytes were cultured under hypoxic conditions (**A**) or stimulated with TNF-α (**B**). Uric acid release into the media and cellular XOD activity were measured.

**Figure 7:**
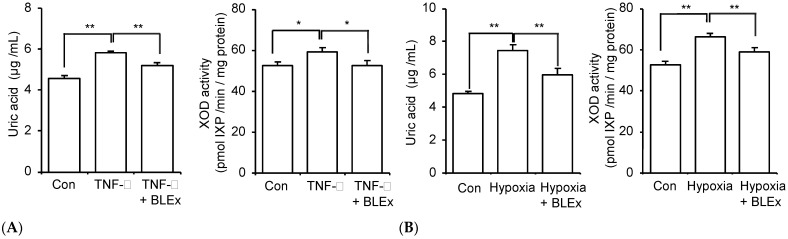
Effect of BLEx on uric acid production by 3T3-L1 adipocytes under TNF-α stimulation and hypoxic stress. Data are means ± SE for 3 independent experiments. Statistical analysis was performed using one-way analysis of variance (ANOVA) and post hoc tests (Tukey–Kramer multiple comparisons). Asterisks indicate significant differences compared to the control at *p* < 0.05 * or *p* < 0.01 **. The 3T3-L1 cells were differentiated into adipocytes along with 100 μg/mL BLEx, and matured adipocytes were then treated with TNF-α (**A**) or 2.5% O_2_ hypoxic conditions (**B**). A: Effect of BLEx on uric acid release into the media and cellular XOD activity. Uric acid release into the media and cellular XOD activity were measured.

Next, we evaluated the effect of BLEx on uric acid production and XOD activity. Figure 5A shows a slight but significant suppressive effect of BLEx on the production of uric acid at 100 μg/mL. A suppressive effect of XOD activity was not observed. Allopurinol, a representative inhibitor of XOD, did not suppress lipid accumulation, but strikingly suppressed uric acid production (Figure 5B). To evaluate the effect of obesity-related cellular stress on uric acid production and XOD activity, 3T3-L1 adipocytes were treated with 1 and 10 ng/mL TNF-α or cultured in hypoxic conditions with 1%, 2.5%, 5%, and 20% oxygen. TNF-α dose-dependently increased the production of uric acid and XOD activity (Figure 6A). The production of uric acid was stimulated by hypoxic conditions and reached a plateau at 2.5% oxygen (Figure 6B). In contrast, a significant increase in XOD activity was observed at 5%, but not at 2.5% and 1% oxygen, compared to that with 20%. We then applied 10 ng/mL TNF-α and 2.5% oxygen for evaluating BLEx. As shown in Figure 7, BLEx alleviated the increase in uric acid production and XOD activity stimulated by TNF-α and hypoxia.

### 2.6. Direct Inhibitory Effect on XOD Activity

**Figure 8:**
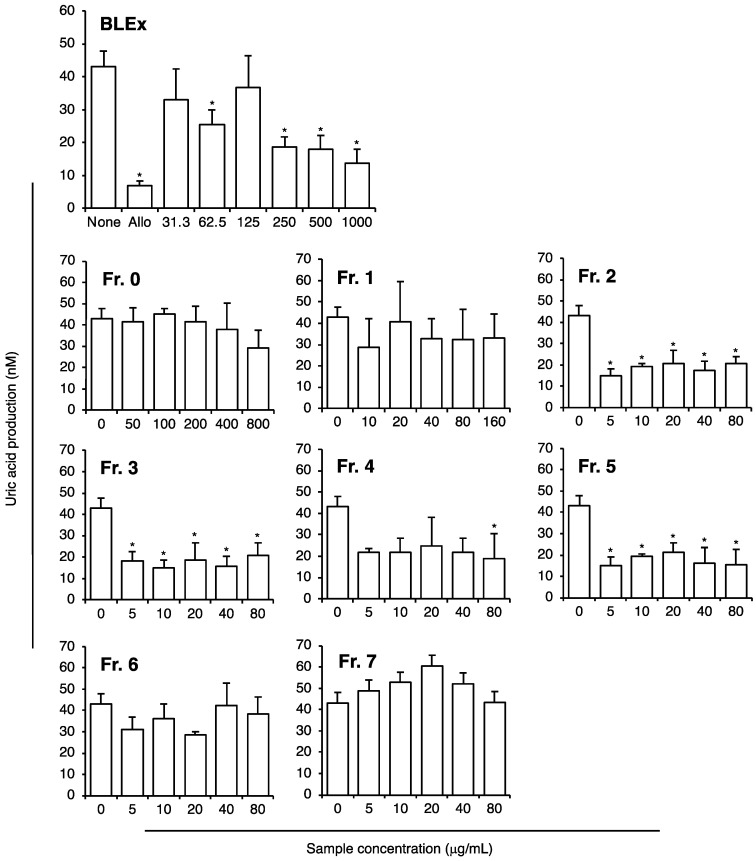
Direct effect of BLEx and its fractionated samples on XOD activity. Data are means ± SE for 3 independent experiments. Statistical analysis was performed using one-way analysis of variance (ANOVA) and post hoc tests (Tukey–Kramer multiple comparisons). Asterisks indicate significant differences compared to the control at *p* < 0.05. XOD activity was evaluated by the conversion of xanthine into uric acid with or without BLEx or its fractionated samples at the indicated concentrations. As the positive control, inhibition of XOD activity by allopurinol (1 mM) is shown in the panel of BLEx.

Finally, the direct effect on XOD activity was evaluated using the purified XOD enzyme in vitro (Figure 8). BLEx clearly inhibited XOD activity in a dose-dependent manner from 62.5 to 1000 μg/mL and the IC50 was estimated to be 100.0 μg/mL. To identify the responsible fractions, the effect of each fraction (Fr. 0–Fr. 7) on XOD activity was evaluated. Here, the concentrations of each sample were set based on the yield of the fractionation experiment. For instance, the yield of Fr. 0 was 52.83%, and the concentration of Fr. 0 was set between 50 and 800 μg/mL. The results showed that Fr. 2, 3, 4, and 5 have a significant ability to suppress the activity of XOD at concentrations corresponding to the IC50 of BLEx. Fr. 0 showed a moderate effect at the highest concentration, and Fr. 6 and 7 showed no effect. We found that Fr. 2, 3, and 4 showed the ability to inhibit XOD. As the proanthocyanidin content in these fractions was below BLEx, the polyphenol composition was analyzed using HPLC (Table 2). The major polyphenol present in Fr. 2 and Fr. 3 was chlorogenic acid, and that in Fr. 4 was rutin. Epicatechin and catechin were the second major components in Fr. 2, 3, and Fr. 4, respectively.

## 3. Discussion

Plant extracts rich in polyphenols are attracting increasing attention as materials for the reduction of adipogenesis in adipocytes [22]. BLEx is also rich in polyphenols such as chlorogenic acid, rutin, and proanthocyanidins, and accumulating data show their anti-lifestyle disease effects, including obesity. Here, we showed the inhibitory effect of BLEx on adipogenesis, and the specific degree of polymerization of proanthocyanidins (Fr. 6) was attributed to this effect. Chlorogenic acid has been shown to exert a body fat-reducing effect [23,24], whereas it promotes adipogenesis of 3T3-L1 at 20 μM [25]. Rutin, a major polyphenol of BLEx, is also a positive regulator of adipogenesis in 3T3-L1 cells at 3 μM [26] but inhibits adipogenesis at high concentrations [27]. Moreover, quinic acid has the ability to inhibit adipogenesis of 3T3-L1 cells at 96 μg/mL [28], whereas 100 μg/mL BLEx includes 18.7 μg/mL, which is below the effective concentration. Taken together, these results suggest that the major polyphenol monomers and organic acids of BLEx did not contribute to the antiadipogenic effect of BLEx in 3T3-L1 cells.

BLEx and Fr. 6 inhibited the mRNA expression of *pparg, aP2, hsl,* and *lpl*, which are differentiation markers for the late stage of adipocyte differentiation accompanied by adipogenesis, indicating inhibition of adipocyte maturation. Although a previous study showed that oligomer proanthocyanidins inhibited adipogenesis [29,30,31], Fr. 4, 5, 7, which included proanthocyanidins, failed to show this effect. As shown in Figure 3, we aimed to identify the active fraction and, thus, we set the concentration corresponding to that in 100 μg/mL BLEx. Therefore, the concentrations of Fr. 4, 5, and 7 were 3.98, 8.34, and 3.52 μg/mL, respectively. Compared with previous studies, it is noteworthy that the effective concentration of Fr. 6 was 1.88 μg/mL, which was more than 10 times lower. This finding implies that the specific molecular size of proanthocyanidins included in BLEx has a prominent inhibitory effect on adipogenesis. As the mean degree of polymerization (mDP) of proanthocyanidins in Fr. 6 was 7.55, a specific mDP might be required for the action of proanthocyanidin. In addition, specific units of proanthocyanidin might have contributed to this effect. Proanthocyanidins in BLEx are composed of (+)-catechin and (-)-epicatechin as the terminal units and (-)-epicatechin, procyanidin A-2, and cinchonains Ia and Ib as the extension units, and that there are at least dodecamers with A-type linkages and phenylpropanoid moieties [10]. Although Table 1 also shows the occurrence of A-type linkages and cinchonain units in BLEx, the characteristic values in Fr. 6 were not observed. We will thus focus on the relationship between proanthocyanidin structure and its effect in future studies.

The effect of norepinephrine-induced lipolysis was evaluated, as shown in Figure 4. Here, we added BLEx or Fr. 6 when the cells matured to negate their effect on the differentiation and maturation process. Norepinephrine increases cAMP levels through β-adrenoreceptors, leading to the activation of HSL and lipolysis, which can be evaluated as glycerol or free fatty acid release [32]. BLEx, but not Fr. 6, stimulated lipolysis under norepinephrine stimulation. Therefore, BLEx was considered capable of suppressing adipogenesis and promoting lipolysis, and only the former effect was attributed to proanthocyanidin in Fr. 6. Although this is the first report on the lipolytic effect of BLEx, proanthocyanidins, chlorogenic acid, and quinic acid, which are major compounds of BLEx, have been shown to promote lipolysis [28,33]. Although Figure 4 shows that the Fr. 6 failed to promote lipolysis, several fractions may synergistically or additively contribute to this effect because their concentration used here was lower than that used in previous reports [28,33].

Obesity, especially visceral fat accumulation, is closely correlated with the deterioration of uric acid metabolism, such as an increase in serum uric acid levels [34]. Uric acid at 5 mg/dL increased lipid accumulation in adipocytes derived from human bone marrow-derived mononuclear cells [35]. As shown in Figure 1, we detected approximately 5 μg/mL (=0.5 mg/dL) of uric acid in the cultured media of 3T3-L1 maturated adipocytes. Moriya and Satoh also detected approximately 3 mM almost equal to 0.04 mg/dL uric acid in 3T3-L1 adipocytes in their study [36]. Moreover, allopurinol, an XOD inhibitor, failed to inhibit lipid accumulation in the present study, indicating that 3T3-L1 adipocytes are capable of producing uric acid, which is not an autocrine factor that promotes adipogenesis (Figure 5). Therefore, it is convincing that BLEx had the ability to inhibit adipogenesis, whereas the effect on uric acid production and XOD activity was faint under steady-state conditions.

Hypertrophy and hyperplasia of adipocytes in the obese state evokes an inflammatory response and hypoxic stress in adipose tissue, leading to the induction of metabolic disorders via an imbalance of adipokines. In this context, TNF-α is a representative inflammatory adipokine responsible for the onset of lifestyle-related diseases [37,38,39]. Hypoxia induces stabilization of the oxygen sensor protein hypoxia inducible factor (HIF)-1α, which forms a heterodimer with HIF-1β. This dimer HIF-1 transactivates XOD [40,41]. In fact, hypoxia stimulates uric acid production from 3T3-L1 adipocytes [42]. Therefore, the present data shown in Figure 6, showing an increase in uric acid production and XOD activity under hypoxia, were consistent with previous reports. Febuxostat, an XOD inhibitor, attenuates stress-induced hyperuricemia and inflammatory parameters, including TNF-α mRNA expression in the adipose tissue [43], indicating the involvement of uric acid in obesity-related inflammation. Inversely, to the best of our knowledge, this is the first report showing that TNF-α directly promotes uric acid production and XOD activity in 3T3-L1 adipocytes, indicating a positive feedback loop of TNF-α and uric acid production. Therefore, hypoxia and TNF-α are common stimuli in that they induce XOD activity. Although the signaling pathway responsible for TNF-α induced uric acid production is unclear, we have previously shown the inhibitory effect of BLEx on TNF-α-induced insulin resistance in C2C12 myoblasts, in which TNF-α signaling was blocked by BLEx [44]. Prevention of TNF-α signaling may thus partially contribute to the inhibition of TNF-α-induced XOD activation and uric acid promotion by BLEx. These data indicate that BLEx is capable of preventing excess uric acid production from adipocytes under obesity-related hypoxic and inflammatory stresses.

As BLEx inhibited both hypoxia and TNF-α-induced uric acid production and XOD activity, it is reasonable to assume that BLEx has the ability to prevent XOD activity. XOD is a predominant target for patients with gout, and its inhibitors, such as febuxostat and allopurinol, have been applied clinically for the reduction of serum uric acid. As shown in Figure 5, allopurinol strongly reduced uric acid production in 3T3-L1 adipocytes, thus verifying the experimental protocol. On the contrary, no significant effect was observed for any fraction. This result implies that several different fractions contributed to the inhibition of uric acid production. As shown in Figure 8, direct suppression of XOD activity might explain the suppressive effect of BLEx on uric acid production from adipocytes. Although Fr. 6, including highly polymerized proanthocyanidin, was responsible for the suppressive effect of lipid accumulation in adipocytes, it failed to inhibit XOD activity. Moreover, an inhibitory effect of XOD was observed in Fr. 2, 3, 4, and 5. Therefore, it is reasonable to consider that BLEx contains distinct compounds with the ability to suppress lipid accumulation and uric acid production in adipocytes. We confirmed that the mDP of proanthocyanidins in Fr. 4 and 5 were 5.74 and 6.05, respectively, which was smaller than Fr. 6 and 7. This observation is consistent with a report in which an inverse correlation was observed between the degree of polymerization and the suppressive effect of proanthocyanidins on XOD [20]. Interestingly, Fr. 2 and 3 inhibited XOD activity. As the inhibition of XOD was observed in proanthocyanidins as well as in chlorogenic acid, rutin, catechin, and epicatechin [20,45,46,47], it is reasonable to assume that these compounds contribute to the inhibition of XOD activity in a complex manner. The content of these polyphenols showed great variation among cultivars [48,49]; therefore, it is assumed that ability of antiadipogenic and XOD inhibitory activities might differ among cultivars. In particular, rabbiteye, a cultivar used in this study, is known to have relatively high proanthocyanidins content and might have high antiadipogenic inhibitory activity [50,51]. In addition, the polyphenol content varies depending on the cultivar stage. Kai et al. showed that the cytotoxic effect on adult T-cell leukemia cells fluctuates seasonally, and this effect is also likely to be dependent on the content of proanthocyanidins [52,53].

## 4. Materials and Methods

### 4.1. Materials

BLEx was prepared from *Vaccinium virgatum* Aiton; Kinisato 35 Gou harvested in Miyazaki prefecture in Japan. Blueberry leaf (100 g) was extracted with 10 L of hot water (95 °C) for 30 min. Next, the extract was filtered by 300 μm nylon mesh and freeze dried (FD-10BU-SS, Nihon Techno Service, Co., Ltd., Ushiku, Japan) sample was kept in −30 °C before experiments. DMEM, penicillin–streptomycin–amphotericin B mixed solution, dexamethasone, and calcium pantothenate were purchased from Fujifilm Wako Pure Chemical (Osaka, Japan). D-Biotin, fetal bovine serum, and Oil Red O were purchased from Sigma-Aldrich (St. Louis, MO, USA). Isobutylmethylxanthine was purchased from Calbiochem (San Diego, CA, USA). Calf serum was purchased from Thermo Fisher (Waltham, MA, USA).

### 4.2. Cell Culture

The 3T3-L1 cells were purchased from the Japanese Collection of Research Bioresources Cell Bank (IFO50416, Osaka, Japan). These cells were maintained in DMEM supplemented with 10% calf serum containing penicillin-streptomycin-amphotericin B at 37 °C in a 5% CO_2_ atmosphere. For adipocyte differentiation, 3T3-L1 cells were cultured to confluence in DMEM supplemented with 10% fetal bovine serum (FBS). The cells were then cultured with 10% DMEM supplemented with 10 μg/mL insulin, 0.25 μM dexamethasone, 0.5 mM isobutylmethylxanthine, 16 μg/mL calcium pantothenate, and 3.2 μg/mL D-biotin for 48 h for inducing their differentiation to adipocytes. Further, the cells were cultured in maturation media (same composition as the differentiation medium, without dexamethasone and isobutylmethylxanthine). To accomplish hypoxic conditions with a 1%, 2.5%, and 5% oxygen atmosphere, cells were cultured in a BIONIX hypoxic culture system (Sugiyama-gen, Tokyo, Japan). Cytotoxicity of the BLEx was confirmed by 0.2% trypan blue dye solution exclusion method.

### 4.3. Oil Red O Staining

At the end of the culture, cells were fixed with 4% paraformaldehyde at 4 °C for 1 h. Lipid droplets were stained with 3 mg/mL Oil Red O for 1 h at room temperature. After washing, the dye incorporated into the cells was re-extracted with 2-propanol, and the absorbance of the extracts at 540 nm was measured using SpectraMax ABS (Molecular Devices, San Jose, CA, USA). Lipid accumulation was expressed as relative absorbance compared to the control value.

### 4.4. Real-Time PCR

Cells were washed with PBS, and total RNA was extracted using TRIzol^®^ Reagent (Thermo Fischer, Waltham, MA). Reverse transcription was performed using 1 μg of extracted RNA with the ReverTra Ace qPCR RT kit (Toyobo, Osaka, Japan) according to the manufacturer’s protocol. Briefly, 2 μL of 5 × RT buffer, 0.5 μL primer mix, and 0.5 μL enzyme mix were mixed in a PCR tube and adjusted to 10 μL by adding nuclease-free water. The primer sequences used are shown in Supplementary Table S1. The reaction was incubated at 37 °C for 15 min and then terminated by incubating at 98 °C for 5 min. Real-time PCR was performed using the AriaMX real-time PCR system G8830A (Agilent Technologies, Santa Clara, CA, USA) to evaluate the gene expression of differentiation markers. Briefly, 5 μL QPCR Master Mix (Brilliant III Ultra-Fast SYBR Green QPCR Master Mix; Agilent Technologies), 0.4 μL forward primer (100 nM), 0.4 μL reverse primer (100 nM), 3.5 μL nuclease-free water, and 0.7 μL cDNA were mixed in a 96-well plate (Ina-optica Corporation, Osaka, Japan). The reaction conditions included initial activation for 3 min at 95 °C, followed by 40 cycles of denaturation at 95 °C for 5 s and annealing/elongation at 60 °C for 5 s. After the reaction, the expression of each gene was calculated using Pfaffl’s formula based on the obtained Ct values. The Ct value of *gapdh* was used as the internal control.

### 4.5. Fractionation of BLEx

BLEx (50 g) was suspended in 3 L of distilled water and sonicated at 50 °C for 30 min. The insoluble materials were removed by vacuum filtration, and the filtrate was applied to a column filled with 550 cm^3^ Sephadex LH-20 (GE Healthcare, Chicago, IL, USA). Elution was performed with water containing increasing proportions of methanol (100% water, followed by 20%, 40%, 60%, 80%, and 100% methanol), with a final elution in 60% acetone. We referred to these fractions as Fr. 1–Fr. 7, and the first pass through solution was referred to as Fr. 0. The yield of this fractionation was 92.71%, and the detailed profile of each fraction is shown in Table 3. The eluate was collected, freeze-dried, and dissolved in DMSO for use in subsequent experiments.

### 4.6. Quantification of Proanthocyanidins

For quantifying proanthocyanidins, the fractions were measured using the HCl/butanol method [53]. Each fraction was quantitated from 0.5 mg/mL (for Fr. 0–4) or 0.2 mg/ml (for Fr. 5–7) in methanol. Briefly, 400 μL of the solution was mixed with 3.5 mL of *n*-butanol/ HCl (95:5, *v*/*v*) and 100 μL of 2% (*w*/*v*) NH_4_Fe(SO_4_)_2_·12H_2_O in 2 M HCl, and heated in an oil bath at 105 °C for 30 min. After heating, the reaction mixture was cooled in water for 15 min, and absorbance was measured at 550 nm. The proanthocyanidin content was expressed in mg of cyanidin chloride equivalent/g dry weight.

Polymerized degree of proanthocyanidins: The degree of polymerization of proanthocyanidins was estimated by thiolysis [10]. Fr. 4–8 (2.5 mg) were dissolved in 500 μL ethanol solution consisting of 5% (*v*/*v*) 2-mercaptoethanol, 4% (*v*/*v*) 0.5 M HCl, and 32% (*v*/*v*) H_2_O. The mixture was then heated to 70 °C for 7 h. The resulting thiolytic products were filtered, and the filtrate was injected into the HPLC system. HPLC was conducted on a Q-Exactive mass spectrometry system (GL Sciences, Tokyo, Japan) equipped with a UV–visible detector and an Inertsil ODS-HL column (4.6 mm × 250 mm, 1.9 μm, Thermo Fisher Scientific). The mobile phase conditions were as follows: flow rate, 0.8 mL/min; elution solvent, A (0.1% formic acid in water) and B (acetonitrile); and the gradient program, 10%–28% B from 0 to 10 min, 28–80% B from 10 to 30 min. UV detection was performed at a wavelength of 280 nm. The mean degree of polymerization (mDP) of proanthocyanidins was calculated as follows: mDP = [sum of (2-hydroxyethylthio adducts · *n*) + sum of (free flavan-3-ol · *n*)]/[total free flavan-3-ol], where *n* represents the degree of polymerization of detected flavan-3-ol by thiolysis. Average percentage of A-type bonds = [sum of (thiolysis compounds containing A-type bonds · *n*)/[total free flavan-3-ol]/mDP × 100. Average percentage of cinchonain I unit = [sum of (thiolysis compounds containing cinchonain I unit · *n*)]/[total free flavan-3-ol]/mDP × 100.

### 4.7. Analysis of Polyphenol

Polyphenol analysis was performed using HPLC, according to our previous report [54]. Samples (20 mg) were dissolved in 5 mL of 80% (*v*/*v*) methanol and extracted by ultrasonication (US CLEANER, As One, Osaka, Japan) for 15 min at 37 °C and passed through a 0.22 μm membrane filter (Millipore, Bedford, MA, USA). Each sample underwent HPLC analysis (Prominence LC solution system, Shimadzu, Kyoto, Japan) equipped with an Inertsil ODS3 (Shimadzu) (4.6 mm × 250 mm, 5 μm). The mobile phase conditions were as follows: solvent A, 100% (*v*/*v*) ethanol; solvent B, 20 mM potassium phosphate (pH 2.4); column temperature, 40 °C; detection at 280 nm; flow rate, 1.0 mL/min. The binary gradient was as follows: 85%–68% B (0–12 min), 68% B (12–15 min), 68–55% B (15–20 min), 55–85% B (20 min), and 85% B (20–29 min). Standards of chlorogenic acid, catechin, epicatechin, rutin, and caffeic acid were used to identify and quantify the peaks. Data are shown as the mg of each polyphenol/g weight of each fraction.

### 4.8. Lipolysis Assay

After the differentiation and maturation of 3T3-L1 cells, the cells were treated with BLEx or its fractionated samples for 24 h. The cells were then washed twice with Hank’s buffered saline containing 0.2% bovine serum albumin. The cells were then stimulated with 1 μM norepinephrine for 90 min to release fatty acids into the media. The fatty acids were measured using a commercial kit (LabAssay, Fujifilm Wako) according to the manufacturer’s protocol.

### 4.9. Uric Acid Measurement by HPLC

Uric acid in the culture supernatant was measured using HPLC. The supernatant was treated with 60% perchloric acid to remove proteins and was centrifuged at 10,000× *g* for 5 min at 4 °C. The samples were filtered through a 0.45 μm syringe filter before analysis (Advantec, 13HP045, Tokyo, Japan). HPLC separation was conducted using the Infinity II LC system (Agilent Technologies) equipped with an Intersil ODS-2 column (4.6 mm × 250 mm, 5 μm; GL Sciences) and maintained at 40 °C. The mobile phase was 98% of 74 mM phosphate buffer (pH 2.2), and 2% methanol and uric acid was detected by measuring the absorbance at 284 nm.

### 4.10. XOR Activity

XOR activity was measured as described in a previous report [17]. Briefly, at the end of the culture, cells were lysed using 50 mM Tris-HCl (pH 7.5) containing 150 mM NaCl, 2% Triton X-100, 2 mM EDTA, 50 mM NaF, and 30 mM Na_4_P_2_O_7_ with protease inhibitor cocktail (Nacalai Tesque, Kyoto, Japan) on ice for 30 min. The lysate was centrifuged at 12,000× *g* for 30 min at 4 °C. Samples (50 μL) were mixed with 50 μL of 100 μM pterin in a 96-well black plate (Thermo Fischer) to initiate the enzyme reaction. Isoxantopterin production was monitored for 30 min based on fluorescent intensity at Ex 355 nm/Em 405 nm using the Varioskan Flash (Thermo). XOR activity was calculated as pmol isoxantopterin production/min/mg protein.

### 4.11. XOD Inhibition Assay

The direct inhibitory effect of BLEx and its fractions on XOD activity was evaluated as the conversion of xanthine to uric acid under XOD (from bovine milk, Sigma). Uric acid, xanthine, and allopurinol (Fujifilm Wako) were dissolved in 0.1 M phosphate buffer (PB; pH 8.0). Next, 50 μL PB, 50 μL samples (appropriate concentrations of BLEx, fractions, or 1 mM allopurinol), and 100 μL of 1 mM xanthine were added to a 96-well plate (UV-star, Greiner Bio-One, Kremsmünster, Austria). Finally, XOD was added to a final concentration at 0.1 mU and reacted for 3 min at 37 °C. The production of uric acid was measured as the absorbance at 293 nm using SpectraMax (Molecular Devices, San Jose, CA, USA).

### 4.12. Statistical Analysis

Statistical analysis was performed using Statistical Analysis for Mac ver.3.0 (Esumi Co., Ltd., Tokyo, Japan). The data were analyzed using Student’s *t*-test or one-way analysis of variance (ANOVA) and post hoc tests (Tukey–Kramer multiple comparisons). Differences were considered significant at *p* < 0.05.

## 5. Conclusions

Blueberry leaves are a novel material for the prevention of various lifestyle-related diseases. Here, we evaluated the effect of blueberry leaf hot water extract (BLEx) on adipogenesis and uric acid production in adipocytes. This study revealed that BLEx has the ability to inhibit adipogenesis of 3T3-L1 cells in terms of lipid accumulation and mRNA expression of adipocyte maturation markers. The proanthocyanidin fraction with a mean degree of polymerization of 7.55 (Fr. 6) was responsible for this effect but failed to show lipolytic activity. Maturated 3T3-L1 adipocytes prominently produced uric acid with high XOD activity, which was further increased by hypoxic stress and TNF-α. BLEx prevented the action of hypoxia and TNF-α. Interestingly, allopurinol, a representative inhibitor of XOD, inhibited uric acid production, but not adipogenesis. This implies that adipogenesis and an increase in uric acid during adipocyte differentiation and maturation are independent events. Fr. 6 of BLEx was responsible for the inhibiting adipogenesis, but not for uric acid production and XOD activity. Further, proanthocyanidins, with a relatively low mean degree of polymerization (Fr. 4 and 5), as well as other polyphenolic compounds (Fr. 2 and 3) may contribute to the reduction in uric acid synthesis.

## Figures and Tables

**Table 1:** Structural composition of Fr. 4–7 and hot water extracts calculated by the thiolysis of proanthocyanidins.

Fraction Number	mDP *	A-Type Bond (%)	Cinchonain I Unit (%)	B-Type Bond (%)	Proanthocyanidins (mg/g)
BLEx	5.85	4.5	24.3	71.2	73.0
4	5.74	5.2	61.5	33.3	75.2
5	6.05	14.7	6.6	78.7	222
6	7.55	4.7	5.2	90.1	301
7	12.28	4.4	3.8	91.8	241

* mDP: Mean degree of polymerization (mDP) of proanthocyanidins.

**Table 2:** Polyphenol composition of Fr. 2–4 and hot water extracts.

Fraction Number	Catechin	Epicatechin	Chlorogenic Acid	Caffeic Acid	Rutin
BLEx	13.11	5.50	119.17	1.30	22.20
2	1.95	30.33	278.59	5.56	17.07
3	39.68	106.92	198.01	10.24	36.80
4	59.33	16.72	0.97	1.92	217.13

Data are shown as mg/g weight.

**Table 3:** Fraction profiles of BLEx.

Fraction Number	Eluent	Yield (g)	Yield (%)
0	Pass through	26.42	52.83
1	Water	6.08	12.16
2	Water: MeOH (4:1)	1.35	2.70
3	Water: MeOH (3:2)	0.74	1.48
4	Water: MeOH (2:3)	1.99	3.98
5	Water: MeOH (1:4)	4.17	8.34
6	MeOH	3.77	7.54
7	Water: acetone (2:3)	1.76	3.52
8	Water: acetone (2:3)	0.05	0.10
9	Acetone	0.03	0.06
Total		46.36	92.71

## Data Availability

Data is contained within the article or Supplementary Materials.

## Supplementary Materials

The following are available online at https://www.mdpi.com/article/10.3390/plants10122638/s1, Table S1: Primer sequences used for real-time PCR.
