# Supplementary material for: Vaccinium virgatum Aiton Leaves Extract Suppressed Lipid Accumulation and Uric Acid Production in 3T3-L1 Adipocytes
Source: Plants (Basel). 2021 Nov 30;10(12):2638. doi: 10.3390/plants10122638 (PMC8705443; doi:10.3390/plants10122638)
Supplement: Supplementary file 1 [file plants-10-02638-s001.zip › plants-1462224-supplementary.pdf]

Supplement Table S1 Primer sequences used for real-time PCR

Primer	Sequence (5' to 3')
PPARγ Forward	5'-GGAAGACCACTCGCATTCCTT-3'
PPARγ Reverse	5'-GTAATCAGCAACCATTGGGTCA-3'
aP2 Forward	5'-CCGCAGACGACAGGA-3'
aP2 Reverse	5'-CTCATGCCCTTTCATAAACT-3'
HSL Forward	5'-GGCTCACAGTTACCATCTCACC-3'
HSL Reverse	5'-GAGTACCTTGCTGTCCTGTCC-3'
LPL Forward	5'-CATCGAGAGGATCCGAGTGAA-3'
LPL Reverse	5'-TGCTGAGTCCTTTCCCTTCTG-3'
GAPDH Forward	5'-GTGTACATGGTTCCAGTATGACTCC-3'
GAPDH Reverse	5'-AGTGAGTTGTCATATTTCTCGTGGT-3'

PPARγ; peroxisome proliferator activated receptor γ
aP2; Adipocyte fatty acid-binding protein 2
HSL; Hormone-sensitive lipase
LPL; Lipoprotein lipase
GAPDH; Glyceraldehyde-3-phosphate dehydrogenase.
